# Perceptions of Closeness in Adult Parent–Child Dyads: Asymmetry in the Context of Family Complexity

**DOI:** 10.1093/geronb/gbaa122

**Published:** 2020-08-10

**Authors:** Kirsten van Houdt, Matthijs Kalmijn, Katya Ivanova

**Affiliations:** 1 Department of Sociology/ICS, University of Amsterdam, The Netherlands; 2 Netherlands Interdisciplinary Demographic Institute, The Hague, The Netherlands; 3 Department of Sociology, Tilburg University, The Netherlands

**Keywords:** Affectional solidarity, Family complexity, Multi-actor data, Parent–child relations

## Abstract

**Objectives:**

Multi-actor data show that parents’ and adult children’s evaluations of their relation do not necessarily match. We studied disagreement in parent- and child-reported closeness, comparing parent–child dyads involving separated parents, non-separated parents, and stepparents to shed new light on today’s diverse landscape of adult parent–child relations.

**Method:**

Using data from the Parents and Children in the Netherlands (OKiN) survey, we analyzed closeness in parent–child dyads (*N* = 4,602) comparing (step)parents’ and their adult children’s (aged 25–45) reports. To distinguish directional disagreement (i.e., differences in child- and parent-reported means) from nondirectional disagreement (i.e., the association between child- and parent-reported measures), while accounting for absolute levels of closeness, we estimated log-linear models.

**Results:**

All types of parents tend to report higher levels of closeness than their children. Whereas parental overreport is more prevalent among biological father–child dyads than among biological mother–child dyads, we found no differences between biological dyads and stepdyads. The association between children’s and parents’ reports is higher among dyads involving stepmothers or married mothers than among those involving separated mothers and (step)fathers.

**Discussion:**

The intergenerational stake (i.e., parental overreport) is not unique to biological parent–child relations. Instead, patterns of disagreement seem most strongly stratified by gender.

When studying adult parent–child relationship quality (e.g., their level of closeness), the literature on intergenerational solidarity typically relies on the report of only one actor, either child or parent. Yet, children’s and parents’ reports do not necessarily match: Differences in parent- and child-reported closeness are well documented in the literature (e.g., Aquilino, 1999; Birditt et al., 2012; Steinbach et al., 2017). When examining dyadic disagreement, we can think in terms of *directional* disagreement (i.e., the difference between child- and parent-reported means) and in terms of *nondirectional* disagreement (i.e., the association between child- and parent-reported measures). These parameters do not necessarily go in tandem and have their own theoretical implications.

Directional disagreement is central to the “intergenerational stake” literature (e.g., Bengtson and Kuypers, 1971; Giarrusso et al., 2004), which revolves around the consistent finding that, on average, parents see the relation to their adult children in a more favorable light than vice versa. To explain this finding, which is considered unique to the parent–child relation, research has focused on systematic differences between the generations. Possible explanations propose that parents’ perceptions are shaped by motivations of continuity (Bengtson and Kuypers, 1971), returns to parental investments (Giarrusso et al., 1995), or social norms (Mandemakers and Dykstra, 2008).

Nondirectional disagreement is related to the degree of association between parent- and child-reported measures. If parents rate the parent–child relationship more positively than children, this does not necessarily imply that they disagree in *relative* sense. The association between parent- and child-reported measures may still be high if parents systematically report more positively than their children. Conversely, similarity in the means of parents’ and children’s reports does not mean that the association is high, as over- and underreports might cancel each other out. A weak association between parent- and child-reports is usually considered a measurement problem (e.g., Havermans et al., 2015), and its *theoretical* implications have not yet been considered in the literature. Yet, a weak association might indicate that there is lack of consistency between parents’ and children’s frame of reference when evaluating their relationship.

We argue that both these approaches to dyadic disagreement—directional and nondirectional—can shed new light on today’s diverse landscape of parent–child relations. Our aim is to map (dis)agreement in parents’ and adult children’s reports about the level of closeness of their joint relation. We focus specifically on the context of family complexity and compare *directional* and *nondirectional* disagreement in perceptions of closeness along the lines of parental separation, stepparenthood, and parents’ gender.

Increasing numbers of parental separation and repartnering (in this study, both referring to either marital or cohabiting unions) have resulted in a large variety in adult parent–child relations, for example, in terms of biological relatedness (e.g., stepparents or biological parents) or co-residence in childhood (e.g., separated parents with or without physical custody) (Thomson, 2014). First, this context allows us to study to what extent directional disagreement (i.e., parental overreport relative to children’s reports) is standard to parent–child relations. The intergenerational stake literature emphasizes the role of factors that characterize the parent–child relationship—such as biological relatedness, parental investments, and family norms—which are intertwined in the “traditional” family context (Kalmijn et al., 2019). By being the first to compare different types of parent–child ties in which these factors are disconnected, we provide more insight in the mechanisms underlying the intergenerational stake phenomenon.

Second, if there is more ambiguity on what “normal” parent–child relations ought to be—that is, where to compare the relation with—this results in higher levels of nondirectional disagreement. Ties formed by repartnering have been argued to be non-institutionalized (Cherlin, 1978, 2004) and to involve more normative ambiguity (Van Houdt et al., 2018) than those embedded in the well-established first-marriage family. This suggests that there is more ambiguity on how to evaluate parent–child ties involving stepparents or separated parents given the lack of a clear reference. The same might hold for father–child ties, which have been argued to be subject to more weakly established expectations than mother–child ties (Miller, 2010). Comparing the association of parent- and child-reported closeness between different parent–child ties (e.g., stepparents vs biological parents) is a new way to consider the idea of ambiguity in complex families.

Previous studies have described disagreement by comparing means of parent- and child-reported measures. Studies examining *predictors* of disagreement used regression analysis to predict the likelihood or the level of directional disagreement, like difference-score models (Mandemakers and Dykstra, 2008) or multilevel models (Kim et al., 2011). In the present study, we introduce this level of sophistication to the *description* of disagreement of parents’ and children’s reported closeness by using log-linear models. This method perfectly fits the aims of the present study because it allows us to model directional and nondirectional disagreement explicitly. Another important feature is that log-linear models account for ceiling and floor effects. For example, if fathers—in comparison to mothers—more often report higher levels of closeness than their child, this might be the result of the, on average, higher levels of closeness between mothers and children: With a high level of child-reported closeness, there is less potential for the parent to overreport.

Previous studies in the field were based on indirect alter samples (e.g., parents received a questionnaire *after* the anchor respondents provided contact details). This might have led to a selection of parent–child dyads with a relatively high level of consensus (Kalmijn and Liefbroer, 2011). In the present study, we analyze newly collected multi-actor data (Kalmijn et al., 2018) in which Dutch respondents aged 25–45 and their (matched) biological parent(s) and/or stepparent(s) were approached independently and reported on their relation. As a result, the dyads are less selective in terms of contact than surveys based on indirect alter sampling.

Although the majority of studies on the intergenerational stake phenomenon are based on U.S. data, analyses of European data show similar patterns [e.g., German (Steinbach et al., 2017), Dutch (Mandemakers and Dykstra, 2008), and Norwegian (Herlofson, 2013) data]. Therefore, we expect that our findings can be compared to the existing literature and can be generalized beyond the Dutch context.

## Background and Hypotheses

We consider two dimensions of disagreement of parent- and child-reported closeness: Directional disagreement (as reflected by the difference between the parent- and child-reported means) and nondirectional disagreement (as reflected by the association between parent and child reports). Directional disagreement is an absolute measure of difference, whereas nondirectional disagreement is a relative measure. This is similar to studies of intergenerational social mobility (Breen and Jonsson, 2005), in which children have higher levels of education than their parents (high absolute mobility), whereas the association between parents’ and children’s education is still high (low relative mobility). In this section, we consider the theoretical meaning of both dimensions of disagreement and derive hypotheses on differences between types of parent–child ties.

### Directional Disagreement

The literature on directional disagreement started with the observation that parents, relative to their young adult children, overreport affectual and consensual solidarity with their children, as reflected by a higher mean on parent-reported measures (Bengtson and Kuypers, 1971). This difference was originally explained with the idea of different developmental stakes which color parents’ and children’s perceptions: Adult children are concerned with developing a sense of autonomy, and therefore, tend to understate intergenerational affection and overstate intergenerational contrasts. Parents, on the other hand, would see their children as a continuation of themselves, and are concerned with transmitting their values. Therefore, parents tend to overstate affection and understate contrasts in relation to their offspring.

This idea (initially labeled “developmental stake,” and later “intergenerational stake” or “generational stake”) has experienced a number of developments in response to empirical findings (e.g., Birditt et al., 2015; Steinbach et al., 2017), but continues to build on the original idea (parents’ concern with continuity). Giarrusso et al. (1995) add a different explanation by arguing that parents’ perceptions are being colored by a cognitive desire for equity: Parents commonly invest more in their children than vice versa and thus, have a stronger desire for a good parent–child relationship as to legitimize these investments.

Today’s diversity of parent–child dyads raises the question to what extent the abovementioned explanations generalize beyond the biological parent–child relation, embedded in an intact, two-parent family. First, if parents’ overstating of affection and consensus is indeed the result of parents’ perception of their children as “one’s personal extension into the future” (Bengtson and Kuypers, 1971, p. 256), one would expect that this tendency is most prominent when it concerns *biological* offspring. As parents most commonly do not consider their stepchildren as their own (Weaver and Coleman, 2005), their perceptions of these steprelations will also be less susceptible to a desire of continuity.

Second, if parents perceive the relation to their child more positively as to legitimize their parental investments, one would also expect a smaller gap in stepparent–child dyads: Parental investments by stepparents are more heterogeneous (e.g., by variation in duration, timing, and co-residence) and on average lower than those made by biological parents (Ganong and Coleman, 2017). Therefore, we hypothesize that parental overreport is more prevalent among biological parent–child dyads than among stepparent–child dyads (H1).

Furthermore, we explore differences along the lines of parents’ gender. Although the original thesis of parents’ desire for continuity (Bengtson and Kuypers, 1971) is gender neutral, some scholars later predicted that mothers would more strongly overstate closeness due to their higher levels of investment (Giarrusso et al., 1995). However, the empirical evidence does not support this hypothesis. In fact, some studies even find higher levels of overreport among *fathers* (e.g., Shapiro, 2004). By studying different types of parent–child dyads involving the intersection of gender with separation and stepparenthood, we map gender differences in more detail.

### Nondirectional Disagreement

When being asked to evaluate the relationship to their parent/child, respondents compare their own situation to a certain normative model of what a parent–child relation ought to be and to which they anchor their answer (Sudman et al., 1997). One could argue that if there is no clear frame of reference, this leaves more room for dissimilar evaluations of the same relation. Empirically, this would manifest as a weak association between parents’ and children’s reports, that is, nondirectional disagreement. In that sense, the association between parents’ and children’s reports can be considered an indicator for how universal and consistent the normative model is: The weaker the association, the more ambiguous the normative model. Comparing the strength of the association between parents’ and children’s reports over different types of parent–child ties informs us about the level of ambiguity of the normative model.

The idea that ties formed by repartnering are non-institutionalized (Cherlin, 1978, 2004) suggests that, in comparison to the well-established biological parent–child relation, it is more ambiguous what constitutes a “normal” stepparent–child relation. For example, there is less consensus on children’s obligations towards stepparents than towards biological parents (Van Houdt et al., 2018). In addition, stepparent–child ties are less common than biological parent–child ties, and more heterogeneous in terms of timing and context (Van der Pas et al., 2013): Whereas some stepparents have lived with their stepchild throughout the child’s youth, other stepparents have been acquired in later life. Therefore, when evaluating a stepparent–child relation, a normative model might be more difficult to determine (as they are less common) and more variable (given the higher level of heterogeneity) than for biological parent–child ties. As a result, stepparents and children might vary more in the comparisons they make to evaluate their relation. This leads to the hypothesis that parents’ and children’s reports of closeness are more strongly associated among parent–child dyads involving biological parents than among dyads involving stepparents (H2).

To some extent, the same arguments can be made for relations between children and separated parents (in comparison to non-separated parents), which are also less strongly institutionalized (Cherlin, 1978) and more heterogeneous in nature than relationships with non-separated parents. The turbulence and conflict that commonly follows parental separation might lead to more ambivalence and less agreement in perspectives on how close the relation between parent and child is, but also what would be “normal” in that context. Therefore, we hypothesize that the association between parents’ and children’s reports is stronger among dyads involving married biological parents than among dyads involving separated biological parents (H3).

With respect to parents’ gender, father–child relations can be argued to have an ambiguous normative model in comparison to mother–child relations. Whereas the ideal of mothers as warm, involved caretakers is well established, ideals of fatherhood are less consistent. Traditionally, the emphasis has been on the father as the family’s “provider” (Christiansen and Palkovitz, 2001), but more recently, intimacy between fathers and children has become a more prominent part of fatherhood ideals (Miller, 2010). Therefore, we hypothesize that the association between parents’ and children’s reports is stronger among mother–child dyads than among father–child dyads (H4).

## Method

### Data

We analyzed multi-actor data from the *OKiN* survey (Ouders en Kinderen in Nederland; *Parents and Children in the Netherlands*; 2018). This survey contains two samples, adult children (aged 25–45; *anchors*) and their (step)parents (*alters*). The anchor sample was drawn from the population registers of independently living persons born in the Netherlands between 1971 and 1991. It contains an oversample (75%) of persons who grew up with separated (including former marital and cohabiting unions) or widowed parents, and persons who grew up with a stepparent. The sampling strata were defined by the registered address of the child at age 15 and the biological parents and possible new partners. The alter sample was derived from the anchor sample, using the population registers: The biological parents of the adult child and their current partners (i.e., partners registered in the same household at the time of drawing the sample) were selected. The parents were approached independently from the anchors.

The fieldwork was done by Statistics Netherlands (CBS) by means of web-based questionnaires, followed by face-to-face interviews (anchors) or paper-and-pencil questionnaires (alters) in case of initial non-response. This resulted in samples of 6,485 adult children (response rate 62%) and 9,325 (step)parents (response rate 38%).

We analyzed the parent–child dyads in which both the (step)parent and the child participated in the survey. In the parent questionnaire, the anchor child was identified on the basis of the date of birth. Children could have up to four participating (step)parents, hence, children might be represented in multiple parent–child dyads in our data. The final sample (4,602 parent–child dyads) includes 3,032 children, of which the number of participating parents was one (56%), two (37%), three (6%), or four (1%). Table 1 describes the analytical sample in terms of its demographic characteristics and the key variables. There were no missing values.

**Table 1.** Descriptive Statistics of Analytical Sample

	*M*/Proportion	*SD*	Min.	Max.
*Adult children (N = 3,032)*				
Age	33.22	5.53	25	45
Female (ref. male)	0.52			
Children (ref. no children)	0.51			
*Dyads (N = 4,602)*				
Age (parent)	61.70	6.63	30	85
Closeness reported by child	2.71	1.12	0	4
Closeness reported by parent	2.99	1.00	0	4
Type of parent–child dyad				
Biological mother married	0.18			
Biological mother separated	0.28			
Biological father married	0.16			
Biological father separated	0.17			
Stepmother	0.10			
Stepfather	0.12			

### Measures

*Closeness* was measured with the question “How close is the relationship to your [parent/child] currently?,” answered on a 5-point scale from “not close at all” to “very close.”

We distinguished six *types of parent–child dyads*: Dyads involving married biological mothers, separated biological mothers, married biological fathers, separated biological fathers, stepmothers, and stepfathers. Separated and married refers to the union in which the anchor respondent was born. The “married” category includes both married and cohabiting parents. In the questionnaires, stepparent–child dyads were defined as “the current partner of your father/mother” (from the child’s perspective) and “children of your current partner from a previous relationship” (from the stepparent’s perspective).

### Analytical Strategy

We estimated log-linear models of disagreement of parent- and child-reported closeness. Log-linear models have often been used to model patterns of relative homogamy or mobility (e.g., Blackwell and Lichter, 2004; Hout, 1983; Kalmijn, 1991), but they have also been used to model asymmetry in cross-tabulations, like for marriage (hyper- vs hypogamy; Y. Qian and Z. Qian, 2017) and status mobility (upward vs downward mobility; Kye and Park, 2019). Previous studies on the intergenerational stake phenomenon which focused on predicting disagreement used regression analysis, like difference-score models or multilevel models. Given our different aims—describing patterns of disagreement—the log-linear model is a more attractive method for this study as it has a number of important advantages as discussed below.

**Figure 1.**
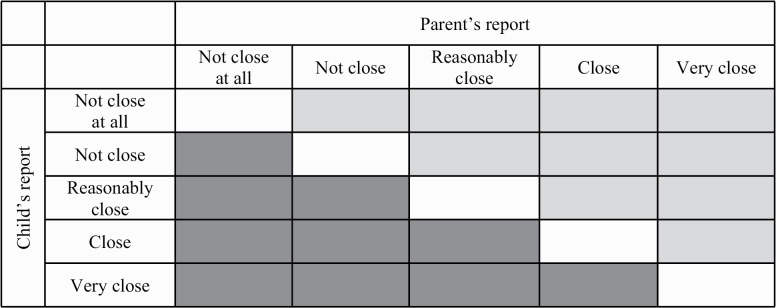
Agreement and disagreement as reflected in a cross-tabulation of closeness. *Note:* White denotes dyads with agreement, light gray denotes dyads with parental overreport, and dark gray denotes dyads with child’s overreport.

The basis of the models is a cross-tabulation of child-reported closeness by parent-reported closeness. The cells on the diagonal represent agreement, the off-diagonal cells represent disagreement, with the cells under the diagonal representing parental overreport and the cells above the diagonal representing child’s overreport (Figure 1). To draw meaningful conclusions about directional and nondirectional disagreement, the models need some additional specifications.

First, the patterns of (dis)agreement we observe are sensitive to the marginal distributions of closeness. Namely, the frequency of any cell is determined by the size of the marginal totals. For example, if many parents feel close to their children, and many children feel close to their parents, we observe a high association as a result of a high level of closeness rather than as a result of agreement per se. This is an important distinction in our case specifically, as we make a comparison between agreement in stepparent–child relationships (involving large heterogeneity in terms of closeness) and biological parent–child ties (which tend to concentrate on the upper part of the closeness distribution). Second, the level of parental overreport is dependent upon the level of closeness itself. Namely, if a parent and a child feel close (i.e., they identify with the upper categories of the scale), there is less potential for parental overreport than if they feel less close.

Log-linear models are particularly suitable for this study as they (a) allow us to model directional and nondirectional disagreement explicitly while (b) taking these two factors (marginal distributions and ceiling/floor effects) into account. The models (formulated in Table 2) predict the number of observations in each cell of the cross-tabulation (see Figure 1), using Poisson regression. The (type of parent–child dyad specific) marginal distributions are modeled with the parameters $\lambda_{ik}^{PT}$ and $\lambda_{jk}^{CT}$. By imposing the constraint that, within types of parent–child dyads, the parameters for the marginal distribution of closeness reported by parents are equal to children’s ($\lambda_{ik}^{PT}=\;\lambda_{jk}^{CT}$), the model accounts for differences in distributions. In this so-called *symmetry* model (Model 1), we estimate the overall association between child-reported and parent-reported closeness by adding the uniform association parameter $\varphi P_{i}C_{j}$, which reflects the degree of nondirectional agreement. In the next step (Model 2), we estimate the odds of parental overreport (cells under the diagonal) in comparison to children’s overreport (cells above the diagonal) with the asymmetry parameter $\lambda^{P_{i}>C_{j}}$ (Fu, 2001), which reflects the degree of directional disagreement.

**Table 2.** Model Specification Log-Linear Models

Model 1	log $F_{ijk}=\;\mu +\;\lambda_{ik}^{PT}+\;\lambda_{jk}^{CT}+\lambda_{k}^{T}+\varphi P_{i}C_{j}+e_{ijk}$	with $\lambda_{ik}^{PT}=\;\lambda_{jk}^{CT}$ if i=j
Model 2	log $F_{ijk}=\;\mu +\;\lambda_{ik}^{PT}+\;\lambda_{jk}^{CT}+\lambda_{k}^{T}+\lambda^{P_{i}>C_{j}}+\varphi P_{i}C_{j}+e_{ijk}$	with $\lambda_{ik}^{PT}=\;\lambda_{jk}^{CT}$ if i=j
Model 3	log $F_{ijk}=\;\mu +\;\lambda_{i}^{PT}+\;\lambda_{j}^{CT}+\lambda_{k}^{T}+\lambda^{P_{i}>C_{j}}+\varphi_{k}P_{i}C_{j}+e_{ijk}$	with $\lambda_{ik}^{PT}=\;\lambda_{jk}^{CT}$ if i=j
Model 4	log $F_{ijk}=\;\mu +\;\lambda_{ik}^{PT}+\;\lambda_{jk}^{CT}+\lambda_{k}^{P_{i}>C_{j}}+\lambda_{k}^{T}+\varphi_{k}P_{i}C_{j}+e_{ijk}$	with $\lambda_{ik}^{PT}=\;\lambda_{jk}^{CT}$ if i=j
Number of dyads in each cell P×C×T (150 cells)		*F*_*ijk*_
Grand mean		μ
Parameter		λ
Uniform association parameter		φ
Parent-reported closeness		*P* with *i* = 0, …., 4
Child-reported closeness		*C* with *j* = 0, …., 4
Type of parent–child dyad		*T* with *k* = 1, …., 6

We studied differences between types of parent–child dyads in two steps. In Model 3, we included an interaction term between type of parent–child dyad ($T_{k}$) and the uniform association parameter ($\varphi P_{i}C_{j}$) to test differences in nondirectional disagreement (H2–H4). In Model 4, we included an interaction term between type of parent–child dyad ($T_{k}$) and the parameter for asymmetry ($P_{i}>C_{j}$) to test differences in directional disagreement (H1).

These models do not allow us to take data clustering (dyads nested in children) into account. However, additional models of directional disagreement (see Supplementary Tables A1 and A2) suggest that adjusting for clustering has little to no effect on the standard errors. Furthermore, the aim of the log-linear models is to describe disagreement in different types of parent–child dyads. As they do not contain any explanatory variables, they also do not include control variables. Yet, the estimation of (a) linear regression models predicting the dyadic difference between parent- and child-reported closeness and (b) logistic regression models (see Supplementary Tables A1 and A2) predicting the likelihood of parental overreport with and without a number of basic control variables—parent’s age, age difference parent and child, parent’s health, and parent’s number of children—indicates that the differences between types of parent–child dyads in directional disagreement are not affected by controlling for these variables in any remarkable way. In addition, to assess to what extent our findings are universal to parents of different age groups and to sons and daughters, we estimated the log-linear models separated by parents’ cohorts, as well as, separated by the child’s gender.

## Results

### Descriptive Findings

Table 3 shows the mean values of parent- and child-reported closeness, by type of parent–child dyad. On average (bottom row), both approach the value of 3, which corresponds to the answer category “close.” Yet, there is a consistent difference between parents’ and children’s reports: On average, parent-reported closeness is 0.28 points higher than child-reported closeness. Although the difference is only a fourth of the standard deviation, it is consistent over the different types of parent–child dyads. Whereas certain parent–child dyads (e.g., the married mother-child dyad) are clearly closer than others (e.g., the stepmother-child dyad), the difference shows little variation over the different types.

**Table 3.** Descriptive Analyses Closeness

	Reported closeness			Overreport		*r*
	Child	Parent	*P*–*C*	Child	Parent	
Biological mother married	3.13	3.33	0.20***	17%	32%	.46***
Biological mother separated	3.00	3.24	0.24***	18%	35%	.55***
Biological father married	2.90	3.21	0.31***	16%	40%	.42***
Biological father separated	2.33	2.74	0.41***	14%	42%	.61***
Stepmother	1.79	2.08	0.29***	17%	40%	.64***
Stepfather	2.47	2.72	0.25***	22%	39%	.54***
Total	2.71	2.99	0.28***	17%	37%	.62***

*Notes:* OKiN data. *N* = 4,602 parent–child dyads.

***p < .01.

To gain insight into similarity on a *dyadic* level, the fourth and fifth column of Table 3 show the prevalence of dissimilar reports, split by parent’s or child’s overreport. Parental overreport is clearly more common. In 37% of the dyads, the parent reported a closer relation than the child, whereas in only 17% of the dyads, the opposite holds. There is some variation over the different types of parent–child dyads in prevalence, as well as, the ratio of parents’ and children’s overreport. Especially in comparison to biological mother–child dyads, parental overreport seems more prevalent among biological father–child dyads and stepmother–child dyads, in both absolute and relative terms.

Lastly, we looked at the correlations between parent- and child-reported closeness, by type of parent–child dyad (last column, Table 3). With an overall correlation of .62, the data reflect the fact that both reports measure the same concept but with a certain level of inconsistency. Furthermore, the correlations illustrate the notion that directional and nondirectional disagreement are different concepts: The types of parent–child dyads with the highest prevalence of parental overreport appear to be dyads among which the reports are also most strongly correlated. This indicates that with less consistency (weaker association), children’s and parents’ overreport cancel each other out (smaller difference between the averages).

### Log-Linear Models

The bottom of Table 4 shows the model fit statistics. Model 2, in which we modeled directional disagreement (i.e., the asymmetry parameter) is a clear improvement over Model 1 (not shown), in which we only modeled nondirectional disagreement (i.e., the association). Model 3, in which we allowed the association to vary between different types of parent–child dyads is again an improvement in fit. Model 4, allowing the asymmetry parameter to vary between types of parent–child dyads, is not a clear improvement (log likelihood improves, but Akaike information criterion and Bayesian information criterion increase). Because the estimates of the interaction pointed at differences between dyads involving biological fathers and mothers but not within these groups, we estimated the variation in the asymmetry parameter more parsimoniously: Instead of estimating the asymmetry parameter separately for six types of parent–child dyads, we estimated it for four types by combining the separated and married biological parents. This alternative specification led to an improvement in fit over the model with a general asymmetry parameter (Model 3).

**Table 4.** Log-Linear Models of Parent- and Child-Reported Closeness

Nondirectional disagreement	Model 2		Model 3		Model 4		Model 5	
	Estimate	*SE*	Estimate	*SE*	Estimate	*SE*	Estimate	*SE*
Uniform association (*φ*)	0.57***	0.02	0.74***	0.08	0.72***	0.08	0.72***	0.08
Interaction type (ref. biological mother married)								
Biological mother separated			−0.15*	0.09	−0.14*	0.09	−0.15*	0.09
Biological father married			−0.22**	0.10	−0.18*	0.10	−0.18*	0.10
Biological father separated			−0.21**	0.09	−0.17**	0.09	−0.18**	0.09
Stepmother			−0.04	0.10	0.07	0.10	0.07	0.10
Stepfather			−0.22**	0.09	−0.20**	0.09	−0.20**	0.09
Pairwise comparisons interaction type								
Biological mother separated vs biological father married			n.s.		n.s.		n.s.	
Biological mother separated vs biological father separated			n.s.		n.s.		n.s.	
Biological mother separated vs stepmother			*		*		*	
Biological mother separated vs stepfather			n.s.		n.s.		n.s.	
Biological father married vs biological father separated			n.s.		n.s.		n.s.	
Biological father married vs stepmother			***		***		***	
Biological father married vs Stepfather			n.s.		n.s.		n.s.	
Biological father separated vs Stepmother			***		***		***	
Biological father separated vs stepfather			n.s.		n.s.		n.s.	
Stepmother vs stepfather			***		***		***	
Directional disagreement								
Asymmetry $(\lambda^{P_{i}>C_{j}})$	0.69***	0.04	0.70***	0.04	0.52***	0.10	0.56***	0.06
Interaction type (ref. biological mother married)								
Biological mother separated					0.06	0.12		
Biological father married					0.29**	0.15		
Biological father separated					0.40***	0.14		
Stepmother					0.27	0.16		
Stepfather					0.11	0.11		
Interaction type (ref. biological mother)								
Biological father							0.31***	0.09
Stepmother							0.23	0.14
Stepfather							0.08	0.13
Pairwise comparisons interaction type								
Biological mother separated vs biological father married					*			
Biological mother separated vs biological father separated					*			
Biological mother separated vs stepmother					n.s.			
Biological mother separated vs stepfather					n.s.			
Biological father married vs biological father separated					n.s.			
Biological father married vs stepmother					n.s.			
Biological father married vs stepfather					n.s.			
Biological father separated vs stepmother					n.s.			
Biological father separated vs stepfather					n.s.			
Stepmother vs stepfather					n.s.			
Pairwise comparisons interaction type (fourfold)								
Biological father vs stepmother							n.s.	
Biological father vs stepfather							*	
Stepmother vs stepfather							n.s.	
Degrees of freedom	32		37		42		40	
Log likelihood	−415.55		−407.24		−401.05		−401.43	
AIC	986.12		993.72		1005.55		996.62	
BIC	895.11		888.48		1005.55		882.86	

*Notes*: AIC = Akaike information criterion; BIC = Bayesian information criterion. OKiN data. *N* = 4,602 parent–child dyads. Model 1 (results not shown) only modeled the uniform association parameter (see Table 2).

*p < .10. **p < .05. ***p < .01.

Model 2 (Table 4) shows that there is a strong association between parent- and child-reported closeness, 0.57 on average. The asymmetry parameter shows that the probability that parents report higher levels of closeness than their child is twice as large as vice versa $\left(e^{0.69}=1.99\right)$.

Model 3, including the interaction between the association and type of parent–child dyad, shows that the association is significantly stronger among dyads involving married biological mothers and stepmothers, than among dyads involving separated biological mothers, biological fathers (separated and married), or stepfathers. This result provides no strong support for a divide between stepties and biological ties (H2) but is more in line with the hypothesis that the association would be stronger among mothers than fathers (H4). At the same time, the weaker association among dyads involving separated biological mothers, in comparison to married biological mothers, provides support for the hypothesis concerning the comparison of separated and married parents (H3), although we did not observe this difference among fathers.

The interaction between the asymmetry parameter and type of parent–child dyad (Model 5) does not provide support for our hypothesis that parental overreport would be less prevalent among stepparent–child dyads (H1). Instead, we found a gender difference among biological parent–child dyads: The chance ratio for overreport is 64 percentage points larger for dyads involving biological fathers than for those involving biological mothers $(e^{0.56}=1.75$ vs $e^{0.56+0.31}=2.39)$.

### Differences by Child’s Gender and Parents’ Cohort

The intergenerational solidarity literature shows that parent–child relations are not only stratified by the parent’s gender but also by the child’s, and their intersection (Willson et al., 2003). Therefore, we estimated separate models for parent–daughter and parent–son dyads (Table 5). The finding that the association is stronger for dyads involving married biological mothers or stepmothers in comparison to other types of parents seems driven by parent–son dyads. The lack of such a difference among parent–daughter dyads seems to be the result of a weaker association among mother–daughter dyads (compared to mother–son dyads). In contrast, the finding that fathers’ overreport is more likely than mothers’ overreport only applies to parent–daughter dyads, not to parent–son dyads.

**Table 5.** Log-Linear Models of Parent- and Child-Reported Closeness by Child’s Gender

Nondirectional disagreement	Daughters		Sons		Parent ≤ 60		Parent > 60	
	Estimate	*SE*	Estimate	*SE*	Estimate	*SE*	Estimate	*SE*
Uniform association (*φ*)	0.60***	0.10	0.85***	0.12	0.95***	0.14	0.62***	0.09
Interaction type (ref. biological mother married)								
Biological mother separated	−0.05	0.11	−0.31**	0.13	−0.40***	0.15	−0.07	0.11
Biological father married	−0.05	0.13	−0.40***	0.15	−0.56***	0.18	−0.08	0.11
Biological father separated	−0.01	0.12	−0.38***	0.13	−0.39**	0.17	−0.13	0.10
Stepmother	0.09	0.14	−0.04	0.16	−0.16	0.17	0.13	0.15
Stepfather	−0.11	0.13	−0.42***	0.14	−0.37**	0.17	−0.24**	0.11
Directional disagreement								
Asymmetry $(\lambda^{P_{i}>C_{j}})$	0.50***	0.09	0.60***	0.09	0.48***	0.09	0.61***	0.08
Interaction type (ref. biological mother)								
Biological father	0.44***	0.13	0.19	0.13	0.02	0.16	0.41***	0.12
Stepmother	0.18	0.20	0.22	0.21	0.16	0.18	0.32	0.24
Stepfather	0.16	0.16	−0.14	0.18	−0.56	0.20	−0.01	0.17
Degrees of freedom	40		40		40		40	
Log likelihood	−300.24		−310.16		−279.95		−326.14	
AIC	680.48		700.33		637.89		732.28	
BIC	791.31		811.16		744.94		824.77	

*Note.* AIC = Akaike information criterion; BIC = Bayesian information criterion. OKiN data. *N* = 2,212 parent–son dyads and 2,390 parent–daughter dyads; *N* = 1,953 dyads involving parent aged ≤60 and 2,649 dyads involving parents aged >60.

**p < .05. ***p < .01.

In addition, we estimated the models separated by parents’ cohort (aged ≤60 vs aged >60, see Table 5). Among dyads involving parents aged 60 or younger, the uniform association is generally stronger, the likelihood of parental overreport is lower, and there are no differences between parents in the likelihood of overreport. Among dyads involving parents older than 60, the association is weaker, there is a higher likelihood of parental overreport, and biological fathers are particularly likely to overreport.

## Discussion

Although research on intergenerational solidarity typically relies on the report by either parent or child, the literature shows that these evaluations do not necessarily match. The current study considered disagreement in parent- and child-reported closeness in different types of adult parent–child dyads, and distinguished directional from nondirectional disagreement. Both these approaches to disagreement have their own theoretical implications and shed new light on today’s diverse landscape of parent–child ties.

First, although the phenomenon of parental overreport (i.e., directional disagreement) has been explained as something unique to the parent–child relationship in the intergenerational stake literature, our findings suggest that this idea needs reconsideration: If parents’ views would be colored by motivations of continuity or justification of their investments (Bengtson and Kuypers, 1971; Giarrusso et al., 1995), one would expect that stepparents have less of such a tendency. Contrary to this expectation, we found that parental overreport is just as common in stepparent–child relations as in biological parent–child relations.

In addition, we found an unexpected gender difference among biological parent–daughter dyads, showing that fathers, more commonly than mothers, report higher levels of closeness than their adult daughters. Although this finding is in line with some previous studies (Shapiro, 2004), the existing theoretical framework provides little guidance in explaining this finding. The notion of parental investment would predict a gender difference in the opposite direction, given the high levels of investment of mothers (Giarrusso et al., 1995). We would explain this finding along the lines of a normative model: The normative model for the father’s role might be more ambiguous than the mother’s given that, during the last decades, intimacy between fathers and children has become a more prominent part of fatherhood ideals (Miller, 2010). Being exposed to a less traditional context and idea concerning father–child relations, children might hold “higher” standards than their fathers when it comes to parent–child closeness. The fact that we found this gender difference among parent–daughter dyads only (not among parent-son dyads) is in line with this idea, as women have been found to expect more active involvement of fathers than men do (Hohmann-Marriott, 2009). This idea of a change in normative models also finds support in the analyses separated by parent’s cohort: Only among the older cohort of parents—raised in more traditional context—fathers are more likely to overreport.

Second, our finding that there is no clear step-biology divide in nondirectional disagreement between parents’ and children’s reports suggests that stepparent–child relations are not generally more ambiguous than biological parent–child relations. It challenges the idea that the non-institutionalized character of steprelations per se would make them ambiguous (Cherlin, 1978), as the differences in association seem to be a more complicated interaction of gender, stepparenthood, and separation. The strongest association can be found in dyads involving married biological mothers or stepmothers. We proposed that a clearer normative model provides a more stable point of reference to which respondents, both parents and children, anchor their answer. This suggests that, in comparison to relations to separated parents and stepfathers, relations to stepmothers and married, biological mothers have clearer normative models, being the opposite endpoints of a spectrum: Relations with a married, biological mother are most commonly strong, relations with stepmothers are most commonly weak (Kalmijn et al., 2019). Our further analyses showed that among dyads involving older parents, the association is weaker, but there are also no differences between mothers and fathers. This could suggest that gender differences in ambiguity have diverged over time (cohort-effect) or converged with age (age-effect), which we cannot disentangle with these data.

With using log-linear models, we introduced a new methodological approach to disagreement in multi-actor data. We showed that accounting for the absolute level of closeness as well as its distribution leads to different conclusions than a purely descriptive approach. For example, the observed high percentage of stepmothers reporting higher levels of closeness than their stepchild appears to be the result of a bottom effect rather than a stronger tendency for overreporting.

Our data and method have a number of limitations, which could serve as opportunities for future research. We did not directly test the mechanisms underlying dissimilar reports. For example, by relating parents’ and adult children’s reports to ideas of what a “normal” parent–child relation entails, future research could provide more insight into the role of normative models. Furthermore, although this study made a big step forward by using independently approached anchor and alter data, an open question remains to what extent families with higher levels of agreement select into participating in surveys about family topics. Lastly, the intergenerational stake literature has been focusing on the description or the determinants of the phenomenon without studying its consequences for families and individuals and our study is no exception. One could argue that from someone considered close, one has certain expectations, such as support in case of need. Yet, if the other perceives the relationship differently, they might not attend to the other’s needs or might be embarrassed by a request for help. Especially in the context of complex families, where family-ties are relatively fragile (Raley and Sweeney, 2020), and in which multiple parent-figures compete for the child’s affection, dissimilar views and expectations could form a risk for the family’s functioning and well-being.

## Supplementary Material

gbaa122_suppl_Supplementary_Material
