# Supplementary material for: Perceptions of Closeness in Adult Parent–Child Dyads: Asymmetry in the Context of Family Complexity
Source: J Gerontol B Psychol Sci Soc Sci. 2020 Aug 10;75(10):2219–29. doi: 10.1093/geronb/gbaa122 (PMC7751165; doi:10.1093/geronb/gbaa122)
Supplement: gbaa122_suppl_Supplementary_Material [file gbaa122_suppl_supplementary_material.pdf]

APPENDIX

The log-linear models that were estimated in this study have two important limitations. First, although our data are clustered (some children are represented in multiple parent-child dyads), the models do not allow for adjusting the standard errors for clustering. Second, the log-linear models do not easily allow for including control variables. Although efforts have been made to develop methodological approaches that solve these problems (Jann & Seiler, 2014), their application mainly speaks to the analysis of intergenerational mobility, and goes beyond the scope of our study. Yet, in order to assess to what extent these limitations color our results, we have estimated a number of alternative models. Predicting (1) the likelihood of parental overreport and (2) the dyadic difference between parent-reported and child-reported closeness, we compare the estimates obtained with/without a number of basic control variables and with/without adjusting the standard errors for clustering. As explained in the main text of this study, we consider the results of the log-linear models more informative with regard to testing our hypotheses, as they allow us to estimate directed and undirected disagreement simultaneously, and take the marginal distributions of closeness into account. Therefore, the models below serve the purpose of a robustness check as they only estimate directed disagreement. In order to account for marginal distributions, the models were controlled for the (dyadic) average level of closeness.

Table A1 shows the results of the logistic regression predicting parental overreport (i.e., parent reports higher level of closeness than child vs. a similar or lower level of closeness). Model 1 includes no control variables and does not account for data clustering. In Model 2, control variables were added. In Model 3, the standard errors were adjusted for clustering. First of all, these models all echo the conclusions that could be drawn from the log-linear models regarding directional disagreement: Biological fathers are more likely to report higher levels of closeness than their child than biological mothers and there are no differences between dyads involving biological parents and stepparents. Furthermore, we can conclude from the comparison of these models that these effects, nor their standard errors and significance are affected by the inclusion of control variables or adjustment for clustering in any remarkable way.

Table A2 shows the results of the linear regression models predicting the dyadic difference between parent- and child-reported closeness (parent's report minus child's report). Again, we observed a higher level of parental overreport among biological father-child dyads. Yet, these models showed a significant difference between dyads involving stepmothers and biological mothers as well. This is something we also observed in our

descriptive results, but not in the log-linear models. This might be explained by the fact that the linear regression model controls for the difference in marginal distributions (stepmother-child dyads are most distant, hence have more potential for overreport) less accurately than the log-linear models. Lastly, from the comparison of these models we can again conclude that the effects, nor their standard errors and significance are affected in any remarkable way by the inclusion of control variables or adjustment for clustering.

To summarize, the results of these robustness checks suggest that the limitations of the log-linear models do not cause any serious bias.

Table A1. Logistic regression predicting parental overreport[a]

	Model 1		Model 2		Model 3[b]	
	Est.	SE	Est.	SE	Est.	SE
Average closeness	-0.31***	(0.03)	-0.30***	(0.04)	-0.30***	(0.04)
Type of P-C dyad (ref. bio. moth.)						
Biological father	0.21**	(0.07)	0.16*	(0.07)	0.16*	(0.07)
Stepmother	-0.12	(0.12)	-0.13	(0.12)	-0.13	(0.12)
Stepfather	0.07	(0.10)	<-0.01	(0.10)	<-0.01	(0.10)
Age parent			0.03***	(0.01)	0.03***	(0.01)
Age difference (P-C)			-0.01	(0.01)	-0.01	(0.01)
Health parent			-0.06	(0.04)	-0.06	(0.04)
No. of children parent			0.10***	(0.02)	0.10***	(0.02)
Constant	0.28*	(0.12)	-1.21***	(0.34)	-1.21**	(0.37)
Log likelihood	-2987.69		-2964.94		-2964.94	

Note. N = 4,602 parent-child dyads.
[a] Parent reported higher level of closeness than child (ref. parent reported similar or lower level of closeness than child). [b] Standard errors adjusted for clustering.
* $p < 0.05$, ** $p < 0.01$, *** $p < 0.001$.

Table A2. Linear regression predicting the difference between parent- and child-reported closeness[a]

	Model 1		Model 2		Model 3[b]	
	Est.	SE	Est.	SE	Est.	SE
Average closeness	-0.15***	(0.02)	-0.14***	(0.02)	-0.14***	(0.02)
Type of P-C dyad (ref. bio. moth.)						
Biological father	0.09**	(0.03)	0.07*	(0.03)	0.07*	(0.03)
Stepmother	-0.11*	(0.05)	-0.12*	(0.05)	-0.12*	(0.05)
Stepfather	-0.05	(0.05)	-0.09	(0.05)	-0.09	(0.05)
Age parent			0.01***	(<0.01)	0.01***	(<0.01)
Age difference (P-C)			-0.01	(<0.01)	-0.01	(<0.01)
Health parent			-0.03	(0.02)	-0.03	(0.02)
No. of children parent			0.05***	(0.01)	0.05***	(0.01)
Constant	0.68***	(0.05)	0.06	(0.15)	0.06	(0.17)
Log likelihood	-6166.84		-6141.27		-6141.27	

Note. N = 4,602 parent-child dyads.
[a] Parent-reported closeness minus child-reported closeness. [b] Standard errors adjusted for clustering.
* $p < 0.05$, ** $p < 0.01$, *** $p < 0.001$.

References

Jann, B., & Seiler, S. (2014). *A new methodological approach for studying intergenerational mobility with an application to Swiss data*. University of Bern Social Sciences Working Paper No. 5. Retrieved from https://boris.unibe.ch/48534/1/jann-seiler-mobility-2014.pdf
